# Sequence-structure-function relations of the mosquito leucine-rich repeat immune proteins

**DOI:** 10.1186/1471-2164-11-531

**Published:** 2010-09-30

**Authors:** Robert M Waterhouse, Michael Povelones, George K Christophides

**Affiliations:** 1 Department of Genetic Medicine and Development, University of Geneva Medical School, 1 rue Michel-Servet, 1211 Geneva, Switzerland; 2 Swiss Institute of Bioinformatics, 1 rue Michel-Servet, 1211 Geneva, Switzerland; 3 Division of Cell and Molecular Biology, Department of Life Sciences, Imperial College London, SW7 2AZ London, UK

## Abstract

**Background:**

The discovery and characterisation of factors governing innate immune responses in insects has driven the elucidation of many immune system components in mammals and other organisms. Focusing on the immune system responses of the malaria mosquito, *Anopheles gambiae*, has uncovered an array of components and mechanisms involved in defence against pathogen infections. Two of these immune factors are LRIM1 and APL1C, which are leucine-rich repeat (LRR) containing proteins that activate complement-like defence responses against malaria parasites. In addition to their LRR domains, these leucine-rich repeat immune (LRIM) proteins share several structural features including signal peptides, patterns of cysteine residues, and coiled-coil domains.

**Results:**

The identification and characterisation of genes related to *LRIM1 *and *APL1C *revealed putatively novel innate immune factors and furthered the understanding of their likely molecular functions. Genomic scans using the shared features of *LRIM1 *and *APL1C *identified more than 20 *LRIM*-like genes exhibiting all or most of their sequence features in each of three disease-vector mosquitoes with sequenced genomes: *An. gambiae*, *Aedes aegypti*, and *Culex quinquefasciatus*. Comparative sequence analyses revealed that this family of mosquito *LRIM*-like genes is characterised by a variable number of 6 to 14 LRRs of different lengths. The "Long" LRIM subfamily, with 10 or more LRRs, and the "Short" LRIMs, with 6 or 7 LRRs, also share the signal peptide, cysteine residue patterning, and coiled-coil sequence features of LRIM1 and APL1C. The "TM" LRIMs have a predicted C-terminal transmembrane region, and the "Coil-less" LRIMs exhibit the characteristic LRIM sequence signatures but lack the C-terminal coiled-coil domains.

**Conclusions:**

The evolutionary plasticity of the LRIM LRR domains may provide templates for diverse recognition properties, while their coiled-coil domains could be involved in the formation of LRIM protein complexes or mediate interactions with other immune proteins. The conserved LRIM cysteine residue patterns are likely to be important for structural fold stability and the formation of protein complexes. These sequence-structure-function relations of mosquito LRIMs will serve to guide the experimental elucidation of their molecular roles in mosquito immunity.

## Background

Disease-vector mosquitoes transmit some of the most devastating diseases of humankind including malaria, dengue, and filariasis. The ability of different mosquito species to transmit these and other pathogens varies greatly and much of this variation in vectorial capacity can be attributed to the success or failure of the mosquito immune system to recognise and eliminate the pathogen. The availability of complete mosquito genome sequences has facilitated both large-scale and targeted experiments, which together with hypotheses generated from extensive comparative genomic analyses have driven dramatic advances in the understanding of vector biology. These studies have revealed key components and underlying mechanisms that constitute the dynamically evolving repertoire of the mosquito's systemic and local epithelial immunity [1].

Recent genetic and biochemical studies of immune system responses to malaria parasite infections in *An. gambiae *have linked three major antiparasitic factors together in a complement-like pathway that mediates parasite killing [2,3]. Two of these factors, LRIM1 and APL1C, are leucine-rich repeat (LRR) containing proteins that form a disulphide-bridged complex that interacts with the third factor, thioester-containing protein 1 (TEP1) a complement C3-like protein. The LRR proteins were initially identified as putative immune factors through microarray studies in *An. gambiae *[4]. Leucine-rich repeat immune protein 1 (LRIM1) was highly upregulated during infection with the rodent malaria parasite, *Plasmodium berghei*, and RNAi-mediated silencing of *LRIM1 *resulted in prominent increases in oocyst numbers, identifying LRIM1 as a key mosquito antagonist of parasite development [5]. A population survey of West African *An. gambiae *mosquitoes mapped the second LRR gene (*Anopheles-Plasmodium-responsive Leucine-rich repeat 1*: *APL1*, also called *LRIM2*) to a genetic locus with major effects on the development and melanization of the human malaria parasite, *P. falciparum *[6]. Laboratory testing of *APL1 *produced similar effects to *LRIM1 *silencing, with significantly increased numbers of developing *P. berghei *oocysts.

Similarly to the effects of silencing *LRIM1 *and *APL1*, knocking down *TEP1 *led to dramatically increased numbers of developing *P. berghei *oocysts [7]. TEP1 binds to bacterial surfaces promoting phagocytosis [8,9] and to the surface of invading ookinetes resulting in their lysis or melanization [7], resembling the roles of vertebrate complement factors. Testing orthologues of all three factors in the non-vector mosquito, *An. quadriannulatus *species A, identified them as key factors in the lysis and melanization responses that these mosquitoes naturally mount against malaria parasites [10]. *LRIM1*, *APL1*, and *TEP1 *are also important in mediating *An. gambiae *immune responses to infections with the rodent malaria parasite, *P. yoelii *[11]. The *An. gambiae APL1 *genomic locus in fact encompasses three distinct genes (*APL1A*, *APL1B *and *APL1C*) of which only the product of *APL1C *acts as a *P. berghei *antagonist [12]. While APL1C functions in immunity against rodent malaria parasites, the *APL1A *gene product protects *An. gambiae *against *P. falciparum *[13]. The LRIM1/APL1C protein complex circulates in the mosquito hemolymph where it is shown to interact with the processed form of TEP1 and promote its subsequent localization on the surface of midgut-invading *P. berghei *parasites [2,3]. In the absence of the LRIM1/APL1C complex, the processed form of TEP1 is found sequestered on self tissues [2]. These studies established the cooperative roles of three key parasite antagonists in the mosquito hemolymph functioning as a complement-like system to achieve targeted pathogen elimination.

LRIM1 and APL1C exhibit several common protein sequence features in addition to their LRRs including signal peptides, patterns of cysteine residues, and coiled-coil domains that identify them as founding members of a family of related mosquito LRR proteins. The relative positioning of only a few key amino acids defines the structural integrity of both LRR and coiled-coil domains, tolerating high levels of sequence variation that may obscure homologous sequence relationships within the superfamily of LRR-containing proteins. This is in contrast to the relatively well-defined family of thioester-containing proteins, which despite the elevated amino acid substitution levels [14] and exceptional allelic polymorphism [15] exhibited by TEP1, form a distinct clade within a superfamily including the pan-protease inhibitors, α-macroglobulins, and the vertebrate complement factors [16,17].

Here we present the results of a comprehensive computational comparative analysis of the sequence characteristics that define the family of LRIM proteins in three disease-vector mosquito species. Predicted structural features suggest an architecture that confers diverse recognition receptor properties with the ability to form multimeric complexes and interact with other components of the mosquito immune system.

## Results

### The LRIM Family

**Figure 1:**
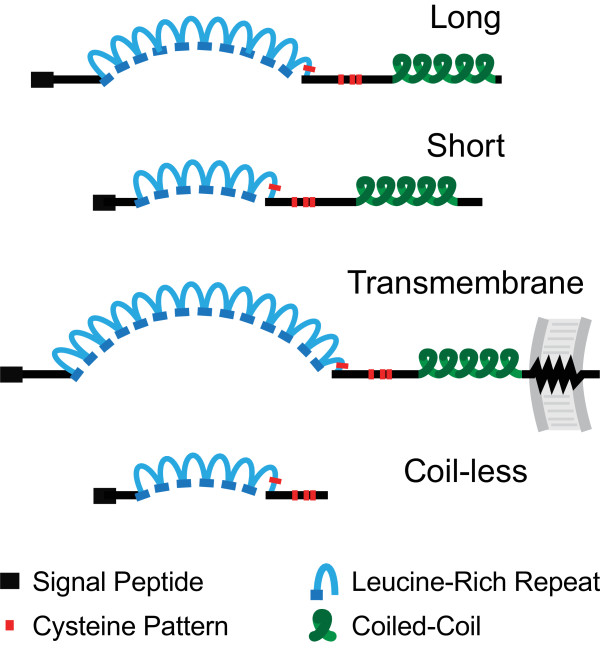
**The identified mosquito LRIM proteins can be classified into four distinct subfamilies**. LRIM family members that match all the defining features are classified as Long LRIMs with 10 or more LRRs, and Short LRIMs with 6 or 7 LRRs. Those exhibiting the defining features but which are additionally predicted to contain a C-terminal transmembrane domain are classified as TM LRIMs, and those lacking only the coiled-coil domain are termed Coil-less LRIMs. The repeating LRR units form a horseshoe-like structure where the short beta-strands form a parallel beta sheet on the concave face of the arc and the linking helices or turns lie on the convex face.

The LRR superfamily is made up of LRR-containing proteins with a variety of domain architectures such as the transmembrane Toll receptors with their intracellular Toll-Interleukin Receptor (TIR) domains. Over 180 LRR superfamily members are found in the predicted proteomes of each of the three mosquitoes as recognised through their InterPro 'Leucine-rich repeat' (IPR001611) annotations. The search for LRR-containing genes with sequence features most closely resembling *AgLRIM1 *and *AgAPL1C *employed a combination of approaches and identified 24 *An. gambiae*, 29 *Ae. aegypti*, and 30 *Cx. quinquefasciatus LRIM*-like genes (see additional file 1). Their encoded proteins exhibit all or most of the key characteristics of *Ag*LRIM1 and *Ag*APL1C: the signal peptide, the LRRs, the patterns of cysteine residues, and the coiled-coils. However, no related genes with these defining characteristics were identified in any of the other representative insect genomes (fruitfly, honey bee, or body louse). The mosquito LRIMs with all the key sequence features can be grouped into the "Long" subfamily with 10 or more LRRs that includes *Ag*LRIM1 and *Ag*APL1C, and the "Short" LRIMs with only 6 or 7 LRRs (Figure 1). Additional related genes include the "TM" LRIMs with a predicted C-terminal transmembrane region, and the "Coil-less" LRIMs that exhibit the characteristic sequence signatures but lack the C-terminal coiled-coil domains.

The principal sequence characteristics of the LRIM family members allow inferences to be made regarding their likely structural architectures, with their common feature of a variable-length LRR domain. The crystal structures of several LRR-containing proteins reveal that each repeat consists of a short beta-strand and a helix or beta-turn where the strands form a parallel beta sheet on the inner face of a horseshoe-like structure whereas the helices or turns lie on the outer face. Structure determination of the human Toll-Like Receptor 3 (TLR3) LRR ectodomain confirmed the horseshoe-like fold, and enabled an interaction model to be proposed where the glycosylaytion-free surface could be important for both oligomerisation as well as ligand binding [18,19].These structures suggest that the 6 or 7 LRR-containing LRIMs are likely to form a shallow arc while those with more LRRs may curve into more extended horseshoe-like structures (Figure 1).

**Figure 2:**
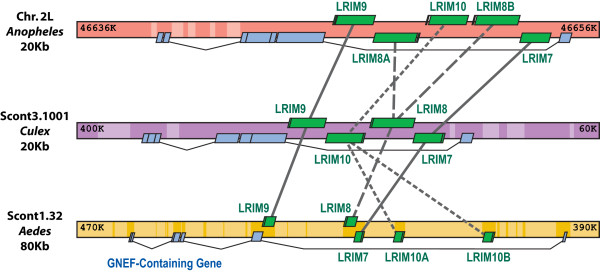
**Orthologous genomic clusters of mosquito Short *LRIM *genes**. *Anopheles gambiae *(red) chromosome (Chr), and orthologous *Culex quinquefasciatus *(purple) and *Aedes aegypti *(yellow) supercontigs (Scont) are depicted with *LRIM *genes (green), a guanine nucleotide exchange factor (GNEF) containing gene (blue), and repeat regions (light shading). Comparative sequence analyses reveal duplications (dashed lines) of *LRIM8 *in *An. gambiae *and of *LRIM10 *in *Ae. aegypti*, while *LRIM7 *and *LRIM9 *remain as single-copy orthologues. Some shuffling of *LRIM *gene orders and orientations has occurred, and the accumulation of repetitive elements has left the *Ae. aegypti *region ~4-fold larger.

Comparative sequence analyses of the mosquito *LRIM*-like genes identified likely orthologous and paralogous relations. This was assisted by examination of orthologous genomic regions (synteny) among the three mosquitoes, which identified clusters of *LRIM *orthologues with local gene duplication and shuffling events. A cluster of short *LRIMs *(*LRIMs 7*, *8*, *9*, and *10*) is in close proximity to a guanine nucleotide exchange factor (GNEF) containing gene found in all three species (Figure 2). Duplications of *LRIM8 *in *An. gambiae *and *LRIM10 *in *Ae. aegypti *have created two paralogous pairs, while *LRIM7 *and *LRIM9 *have remained as single-copy orthologues. The relative location and orientation of *LRIM9 *has remained conserved while *LRIM10 *appears inverted in *An. gambiae*. The *LRIM7-LRIM8 *pair has preserved its head-to-tail orientation in all three species (with the *LRIM8B *paralogue in *An. gambiae*), but in *Ae. aegypti *it has relocated relative to the duplicated *LRIM10*. The genomic span of the orthologous region in *Ae. aegypti *is about four times greater, primarily due to the accumulation of numerous repetitive elements and consistent with the overall ~4.6-fold larger span of synteny regions in *Ae. aegypti *compared to *An. gambiae *[20]. This notable genomic expansion in *Ae. aegypti *is also observed in the *AgAPL1 *cluster, which is located with *LRIM 3*, *4*, and *11 *orthologues between conserved *BRACA2*-like (breast cancer susceptibility protein) and Zinc finger genes. Examining the genomic organisation of *LRIM*-like genes thus reveals events of gene duplication and shuffling that have shaped the evolution of the *LRIM *gene family in mosquitoes.

Several of the identified *LRIM*-like genes have been putatively ascribed immune-related roles from functional studies, suggesting that they too may function in key mosquito immune reactions. These include *AgLRIM4*, a Long LRIM induced in the midgut by *P. falciparum *ookinete invasion [21]; *AgLRIM7 *and *AgLRIM10*, short LRIMs that exhibit transcriptional responses to malaria parasites [21]; and *AgLRIM8B*, a third Short LRIM gene that shows parasite-responsive transcriptional patterns [21] and is downregulated during infections with a Gram-negative bacterium [22]. The Coil-less *AgLRIM17 *gene is also transcriptionally induced in the mosquito midgut upon parasite invasion [21] and was initially identified in the same *An. gambiae *population survey that highlighted the role of *AgAPL1 *in response to *Plasmodium *[6]. Silencing of *AgLRIM17 *revealed that it is an antagonist of both *P. berghei *and *P. falciparum *[21]. In *Ae. aegypti*, the likely *LRIM1 *orthologue is upregulated together with other immune genes following infection with *Wolbachia *bacteria resulting in immune activation and shortened mosquito life spans [23]. Querying results from microarray experiments examining *An. gambiae *transcriptional responses to malaria parasite infections [24] and blood feeding [25] identified at least 18 *LRIMs *with significant changes in gene expression (see additional file 1). Almost all of these *An. gambiae LRIMs *are responsive to blood feeding, while *LRIMs 1*, *4*, *6*, *8A*, *8B*, *10 *and *26 *respond to *P. berghei *infections. In addition, at least 12 *LRIMs *show significantly higher expression in the fat body, the principal insect immune organ, compared to midgut or ovary tissues. The identified *LRIM*-like genes thus form a family of disease-vector mosquito genes that appear to be important effectors in mosquito innate immunity.

### LRIM protein sequence features

**Figure 3:**
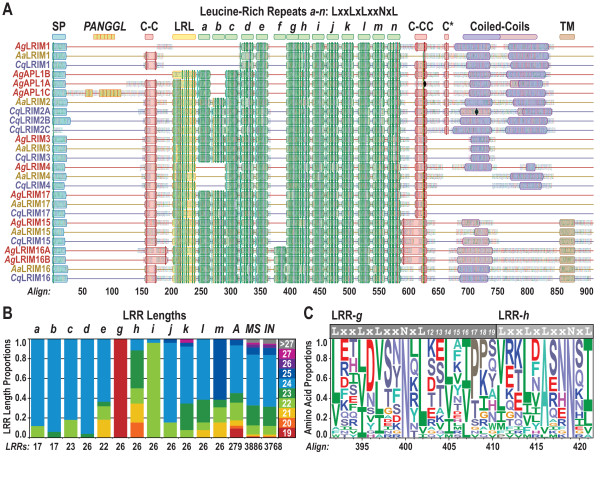
**Characteristic sequence features of the family of mosquito LRIM proteins**. **A**. The annotated multiple sequence alignment of the Long LRIMs (LRIM1-4), the longest Coil-less LRIM (LRIM17), and the TM LRIMs (LRIM15-16) from *Anopheles gambiae *(*Ag*, red), *Aedes aegypti *(*Aa*, yellow), and *Culex quinquefasciatus *(*Cq*, purple). The alignment highlights the defining LRIM features including the signal peptide (SP), patterns of cysteine residues (C-C, C-CC, and C*), leucine-rich leader (LRL), leucine-rich repeats (LRRs *a-n*) and the double and single coiled-coil domains (dark, > 90% propensity, light < 90% propensity). The C-terminal transmembrane (TM) region identifies the TM LRIMs and the PANGGL (Pro-Ala-Asn-Gly-Gly-Leu) amino acid repeat is unique to *Ag*APL1C. Black diamonds indicate positions of a sequence frameshift in the gene encoding *Ag*APL1A and a transposable element insertion in the gene encoding *Cq*LRIM2A. **B**. Examining LRR length variations reveals different constraints on the lengths of sequences connecting the beta-strands that form the LRR horseshoe-like structure. The proportions of LRR lengths are shown for each set of aligned LRRs defined in Figure 3A (excluding those with only 3 or 4 representative sequences) and for those calculated from automated scanning of all 26 LRIMs shown in panel A (*A*), the complete proteomes of each of the three mosquito species (*MS*), and the proteomes of four other insects (*IN*, *Apis mellifera*, *Bombyx mori*, *Drosophila melanogaster*, and *Tribolium castaneum*). **C**. The conservation pattern of amino acid residues of the unusually short LRR-*g *(defined in panel A) is depicted in sequence logo format. The LRR signature is distinguished by the conserved asparagine (*N*) and leucines (*L*) (or the physicochemically similar isoleucines (*I*) and valines (*V*)), and proline (*P*) residues are common at positions 17 and 18 of LRR-*g*.

The repeating nature of both the LRR and coiled-coil domains, together with their tolerance for high levels of amino acid substitutions, present a considerable challenge to multiple sequence alignment algorithms. Nevertheless, stepwise approaches with manual curation of LRIM protein sequence alignments served to identify characteristic features that define the family and help to infer likely sequence-structure-function relationships. These sequence characteristics become clearly discernible on comparing the mosquito proteins most closely related to *Ag*LRIM1 and *Ag*APL1A/B/C (Figure 3): the Long LRIMs (LRIMs 1-4 and APL1), the longest Coil-less LRIM (LRIM17), and the TM LRIMs (LRIMs 15-16).

#### LRIMs are targeted to the mosquito hemolymph

*Ag*LRIM1 and *Ag*APL1C peptide antibodies specifically recognise single protein bands of the predicted sizes in *An. gambiae *hemolymph extracts [3], consistent with the predicted signal peptide sequences that would direct these proteins to be secreted into the mosquito circulatory system. Interestingly, depletion of either transcript blocks secretion of both proteins from hemocytes, indicating that co-expression of these two LRIMs is required for correct formation and secretion of the functional LRIM1/APL1C complex [3]. Apart from one of the *Cx. quinquefasciatus *LRIM2 paralogues (*Cq*LRIM2C, Figure 3A), cleavage sites for signal peptide predictions are found for all the LRIM proteins (see additional file 1). The signal peptide-containing first exon of *CqLRIM2C *is likely obscured by a closely neighbouring transposable element (TE) insertion which may render this gene non-functional. Similarly, a ~4.4 Kb stretch of TE insertions in the coiled-coil encoding region of the *CqLRIM2A *paralogue (Figure 3A) may disrupt the function of this copy, leaving *CqLRIM2B *as the likely functional APL1/LRIM2 orthologue. The signal peptides of the TM LRIMs should direct them towards the secretory pathway, but their hydrophobic C-terminal regions likely anchor them in the cell membrane exposing their LRR-containing ectodomains in a manner similar to the TLRs. The short, ~30 amino acid, intracellular regions of the TM LRIMs have no recognisable sorting or signalling domains, but conserved serine and threonine residues could be potential phosphorylation targets. Secreted into the mosquito hemolymph or exposed on cell membranes (possibly hemocytes), the LRIMs are thus able to circulate widely to sites where an immune challenge may elicit a response.

#### The PANGGL repeat is unique to AgAPL1C

**Figure 4:**
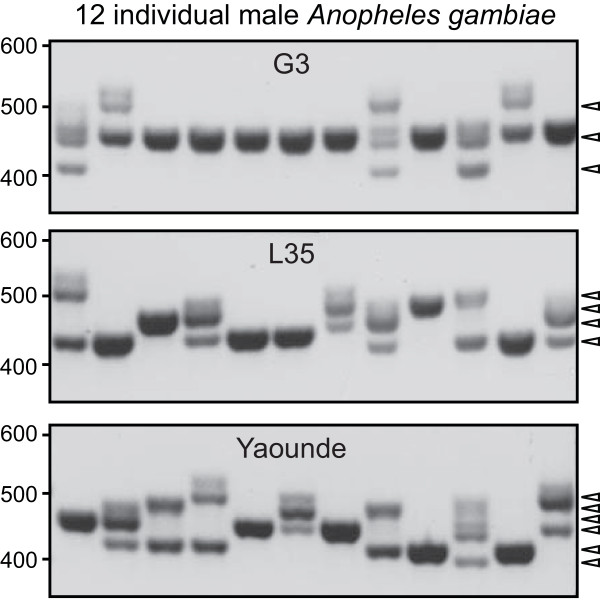
**Variation of *Anopheles gambiae *APL1C PANGGL (Pro-Ala-Asn-Gly-Gly-Leu) repeats in individual male mosquitoes from three laboratory strains**. PCR primers amplify variable-length fragments likely corresponding to up to three, four, and six PANGGL repeat copy-number variations in samples of 12 individual G3, L35, and Yaounde mosquitoes, respectively.

The N-terminal region adjacent to the signal peptide of the *AgAPL1C *gene encompasses multiple repeats of the consensus amino acid sequence Pro-Ala-Asn-Gly-Gly-Leu, PANGGL (Figure 3A). Such repeats are not present in the other LRIM proteins and could not be identified in any other protein-coding genes. Sequencing multiple *APL1C *cDNA clones from laboratory adult male *An. gambiae *mosquitoes indicated the existence of polymorphic *AgAPL1C *alleles encoding variable numbers of PANGGL repeats (Figure 4). The G3 mosquitoes exhibit three major bands likely corresponding to different alleles, while up to six different alleles were identified from individuals of the more recently colonised Yaounde strain. However, the possible structural and functional significance of these PANGGL repeat polymorphisms remains to be determined.

#### LRIMs contain 6 to 14 LRRs of different lengths

The Short LRIMs and the majority of Coil-less LRIMs contain 6 or 7 recognisable LRRs while the LRR domains of the Long, TM, and remaining 3 Coil-less LRIMs are made up of 10 to 14 repeats (see additional file 1). LRR motifs are typically 20 to 30 residues in length and are characterised by an 11-residue consensus defined principally by the spacing of the leucines, *LxxLxLxxNxL *(Figure 3). The LRR pattern tolerates various substitutions with the leucines (*L*) frequently being replaced by valine (*V*) or isoleucine (*I*) and the position of the asparagine (*N*) accepting serine (*S*), threonine (*T*), or cysteine (*C*) replacements. LRRs of the Coil-less LRIMs show frequent *N *replacements while the *N *is ubiquitous among Long LRIM LRRs and highly-conserved among TM and Short LRIMs: *T *replaces *N *in LRR-*k *of the TM LRIMs (Figure 3A), and the Short LRIM5 and LRIM11 each have one LRR where *N *is not maintained. Apart from the LRIM1 orthologues, the LRIM LRR domains are preceded by a leucine-rich leader (LRL, Figure 3A) that resembles the LRR signature sequence but exhibits elevated leucine substitutions and almost never has the characteristic *N*. The similarity suggests that LRLs may form similar strand-helix/turn structures of the more canonical LRRs, but as the first of these repeating structures the LRL sequences are likely to be less constrained. The Short and Coil-less LRIMs also exhibit LRL sequences, but these are less distinct among many Coil-less LRIMs where the LRR consensus sequences are generally less well-defined. Such an irregular type of LRR-like sequence found at the start of the LRR domain is also frequently observed in the sequences of vertebrate TLRs [26]. The last LRR (LRR-*n*) is distinct from the other LRIM LRRs, this LRR is well-conserved with a tryptophan (*W*) or phenylalanine (*F*) consistently replacing the last '*L*' of the LRR consensus followed by a ubiquitously present cysteine residue (*LxxLxLxxNx[WF]xC*). This distinctive terminal LRR pattern is also observed among the Short and Coil-less LRIMs.

Examining the lengths of the LRIM LRRs revealed the exceptionally short LRR-*g*, which is consistently only 19 amino acids long (Figure 3B). The majority of LRIM LRRs are 24 residues in length, which is also the most common LRR length among LRR-containing proteins of mosquitoes and other insects. LRR lengths include the 11-residue consensus identifiable from sequence profiles plus the additional variable-length region before the start of the next consensus, which for LRR-*g *is just 8 residues long (Figure 3C). Although rare, examples of short LRRs with only 19 amino acids have been identified, e.g. Mimivirus protein R380 [27], suggesting that such short LRRs may not necessarily interrupt the complete LRR fold. Proline residues in the LRR variable regions are common in short LRRs where they form part of the convex side of these repeats. Consistent with this observation, proline is the most common residue at positions 17 and 18 of LRR-*g *(Figure 3C). The LRR immediately following the short LRR-*g *exhibits the most variable lengths with LRRs of 20, 21, 22, 23, and 24 amino acids (LRR-*h*) and the third '*L*' of its 11-residue LRR consensus is almost always replaced by a less bulky alanine (*A*) residue. The mosquito *LRIM*-like genes are thus characterised by a variable number of 6 to 14 recognisable LRRs of different lengths, with a subset of LRIMs that exhibit an unusually short LRR of only 19 amino acids.

#### Patterns of LRIM cysteine residues suggest critical disulphide bridging

The comparison of protein sequences most closely related to *Ag*LRIM1 and *Ag*APL1C highlights several well-conserved patterns of cysteine residues (Figure 3A). The leading C-C motif is notably absent from *Ag*LRIM1 and *Ag*APL1B, while the double-cysteine motif (C-CC) is incomplete in *Aa*LRIM2 and missing from the frameshift-disrupted *Ag*APL1A. This frameshift is present in the sequenced *An. gambiae *PEST genome assembly but may not occur in other populations where anti-parasitic effects of *Ag*APL1A have been observed [13]. The double-cysteine motif is consistently replaced by a tyrosine-cysteine motif in the TM LRIMs, and a third solitary cysteine (C*) is conserved in only LRIM1 and LRIM2/APL1 proteins. The leading C-C motif and the double-cysteine motif are also present in all Short and Coil-less LRIMs apart from the three LRIM20 orthologues that lack the leading C-C motif (see additional file 1). Such cysteine patterns are common to many LRR-containing proteins in the regions immediately flanking the LRR domain where they form intramolecular disulphide-bonded caps that stabilise the N-and C-terminal ends of the LRR domains [27]. A double-cysteine motif resembling that found in the LRIMs forms the disulphide-bonded C-terminal cap of the Nogo-66 receptor, a human LRR protein involved in signalling that modulates axon regeneration [28]. This motif contains six cysteines that form three disulphide bridges, suggesting that the LRIM C-CC motif, together with a ubiquitously conserved cysteine residue that immediately follows the last LRR consensus, could allow for the formation of two disulphide bridges to build a stabilising cap.

As well as forming the N-and C-terminal LRR caps, the patterns of cysteine residues may also be important in stabilising LRIM-LRIM interactions as in the case of *Ag*LRIM1 and *Ag*APL1C. Examining the behaviour of *Ag*LRIM1 and *Ag*APL1C using specific antibodies under non-reducing conditions revealed major protein bands of a high molecular weight complex that resolved into expected monomer sizes under reducing conditions [3]. Thus, these two LRIMs form a disulphide-bridged complex, further suggesting that the patterns of conserved cysteine residues that characterise the family of LRIM proteins may be critical to the formation of LRIM complexes. LRR-flanking cysteines have also been implicated in facilitating interactions with other proteins as in the case of mammalian TLR4 and its MD-2 (myeloid differentiation protein) partner required for the recognition of lipopolysaccharide [29]. The MD-2-like family of proteins is expanded in mosquitoes compared to the fruitfly and exhibits six conserved cysteines that may be important in such protein-protein interactions [1,21]. At least one of these MD-2-like proteins in *An. gambiae *shows specificity in regulating resistance to *P. falciparum *[21]. Thus, cysteine residue patterns among LRIM-like proteins may be important for LRR capping as well as for stable interactions both in the formation of LRIM complexes and in the interaction with other protein partners.

#### LRIM coiled-coil domains may facilitate protein-protein interactions

The LRIM1 and APL1/LRIM2 proteins all exhibit a double coiled-coil C-terminal domain (Figure 3A), while the remaining Long LRIMs and all the Short LRIMs exhibit at least one coiled-coil C-terminal region (see additional file 1). Coiled-coil domains can take on a variety of conformations with different helix stoichiometries and orientations [30]. Protein coiled-coils are formed when alpha-helices wrap around each other into stable supercoiled structures of parallel or anti-parallel, homo-or hetero-, dimers or higher order oligomers found in both fibrous and globular proteins. Each of the seven-residue repeats that define the primary structure of coiled-coils gives rise to a complete turn along the alpha-helix, with an amphipathic nature required for supercoil formation. Despite understanding these principles, reliable predictions of coiled-coil domain interactions remain generally unfeasible.

While the predicted monomer sizes of *An. gambiae *LRIM1 and APL1C are ~55 kD and ~80 kD, respectively, the disulphide-bridged complex migrated at ~260 kD suggesting the presence of a functional multimer in the hemolymph [3]. However, given that the observed size changes significantly depending on the resolving power of the gel system used for protein separation (Povelones M, unpublished data), and that coiled-coil containing proteins often exhibit aberrantly slow electrophoretic mobility, it is possible that the complex is assembled from 2-4 LRIM1 or APL1C monomers. Their double coiled-coil domains may facilitate initial associations that bring the proteins together in an orientation to promote the formation of stabilising disulphide bridges. The LRIM coiled-coil domains may therefore play critical roles in facilitating protein-protein interactions, both in the formation of LRIM protein complexes as well as in associating with other components of the mosquito complement-like system.

## Discussion

The identification and feature characterisation of the family of mosquito LRIM-like genes revealed several putatively important sequence-structure-function relations. Notably, their variable LRRs suggest a stable arc or horseshoe-like fold with potential for surface structural plasticity that could facilitate multiple interactions. Interaction properties of LRRs are extremely diverse, exemplified by plant R-proteins that may sense parasites by binding to avirulence proteins or to host proteins that have been perturbed by the presence of parasite effectors [31]. As in the case of the *An. gambiae *LRIM1/APL1C complex, some R-proteins require the formation of homo-or heteromeric complexes to achieve effective activation. LRRs of the related nucleotide-binding leucine-rich repeat (NLR) proteins are believed to sense Pathogen Associated Molecular Patterns (PAMPs) such as flagellin, initiating amino-terminal domain recruitment of downstream effectors such as pro-caspases to trigger immune responses [32]. TLR ectodomains are also composed of LRRs, where in mammals the direct binding of components from infectious agents leads to the intracellular activation of immune responses [33]. In *Drosophila *immunity, the Toll LRRs similarly perform a recognition function but rather than directly sensing PAMPs, the receptor is activated by the cytokine, Spaetzle. The remarkable potential of LRR domains for diverse recognition properties is exploited in the jawless vertebrates, lamprey and hagfish, where LRRs have been shown to form the basis of their adaptive immune system [34]. Combinatorial assembly from a single germline variable lymphocyte receptor (VLR) gene can incorporate repeats from upstream or downstream LRR cassettes to create mature receptors with an incredible diversity of LRRs [35]. Interestingly, lamprey B-subtype VLRs circulate in the blood as disulphide-linked multimers [36], as do *Ag*LRIM1 and *Ag*APL1C in *An. gambiae *hemolymph.

Many thousands of LRR-containing proteins have been found in a wide range of organisms from viruses to eukaryotes where the repeat structures appear to provide versatile recognition and/or interaction functionality often in combination with a variety of other functional, often signalling, domains [37]. Some of the most common include TIR, Nucleotide-Binding Domain (NBD), and protein kinase domains as in the case of many plant R-proteins. Many R-proteins combine a variable number of LRRs with an NBD and an amino-terminal TIR homology region or a coiled-coil domain [31]. Similarly, the identified LRIM-like genes contain a variable number of recognisable LRRs combined with coiled-coils in the Short and Long LRIMs and an additional transmembrane domain in the TM LRIMs. However, none of the LRIM family members encompasses a known signalling domain, suggesting that LRIMs are unlikely to perform immune signalling roles typical of TLRs, NLRs and R-proteins. Instead, LRIMs may operate by sensing and responding to pathogens locally as part of the mosquito complement-like system.

While the LRRs are likely to be responsible for pathogen sensing, the LRIM coiled-coil domains may be important for driving additional interactions. The oligomerisation properties of coiled-coils mean that they can serve as protein-protein interaction domains, as in the case of the SNARE (soluble N-ethylmaleimide-sensitive factor attachment protein receptor) proteins where a four-helix bundle brings three proteins together to facilitate the processes of membrane fusion [38]. Interactions between LRIM coiled-coils may promote favourable orientations of cysteine residues for the formation of stabilising disulphide bridges. This interaction model may be extended to incorporate LRIMs with both double and single coiled-coil C-terminal regions, to form alternative complexes of coiled-coil containing LRIM proteins. As well as promoting structural organisations that allow disulphide bridge formation, the interactions of the coiled-coil domains could direct the specificity of the LRIM-LRIM partnerships, e.g. to ensure the pairing of *Ag*LRIM1 with *Ag*APL1C. Under this model, the Coil-less LRIMs may act as promiscuous family members that are less restricted in their choice of interaction partner. The LRIM coiled-coil domains may also facilitate important interactions with other components of the mosquito complement-like system. The *An. gambiae *LRIM1/APL1C complex interacts with the processed form of TEP1 and promotes its localisation to parasite surfaces [2,3]. Such interactions with components of the complement-like system may be mediated through the coiled-coils of the LRIM complex. In this way, the complex could shield the reactive thioester protein from inadvertent binding to self tissues until sensing of pathogens via the LRIM LRR domains resulted in targeted thioester protein release.

## Conclusions

The known characteristics of the LRR domains of R-genes, NLRs, TLRs, and VLRs, together with functional data from mosquitoes and the interaction properties of coiled-coil domains support the role of LRIM proteins as putative recognition receptors of the mosquito innate immune system. This family of LRIM-like genes appears to be unique and expanded in mosquitoes with more than 20 members in each species but no clearly related genes in other insects. Nevertheless, both LRR and coiled-coil domains are tolerant of high levels of sequence variation that may obscure ancestral relations to genes in other organisms. The 6 to 14 variable-length LRRs that characterise the LRIM proteins may facilitate multiple interactions, which in turn may be augmented by the formation of LRIM protein complexes mediated by coiled-coils and stabilised by disulphide bridges. The appreciation of these relations between key protein sequence and structural features and their likely molecular functions will drive experimental elucidation of their roles in the biology of the mosquito complement-like system and innate immunity.

## Methods

### Gene Prediction

*An. gambiae *LRIM1 and APL1C gene products both have signal peptide sequences followed by a stretch of LRRs. The C-terminal sequences of both these genes exhibit characteristic 7-residue repeats that define the primary structure of coiled-coil domains. A distinctive cysteine-rich pattern can be identified in the hinge region between the LRR and the coiled-coils. These characteristic shared features served as the basis for comparative genomic searches to identify putative LRIM-like genes. Official predicted protein sets for *An. gambiae*, *Ae. aegypti*, *Cx. quinquefasciatus *and the fruitfly, *Drosophila melanogaster*, were scanned for the distinctive cysteine-rich pattern of the hinge region using Perl regular expressions. Hits that also exhibited LRR and coiled-coil domains were then used as seeds for searches of the genomes of the three mosquito species, as well as *D. melanogaster*, *Apis mellifera *(honey bee), and *Pediculus humanus *(body louse). Homology-based gene predictions were then performed for each region using multiple approaches with sequence profiles or individual protein sequences. These predictions were manually examined with available supporting data (e.g. expressed sequence tags) to confirm *LRIM*-like gene models.

### Gene Characterisation

The architectures of the identified LRIM-like genes were analysed using InterProScan [39], REP [40], PCOILS [41], Marcoil [42], and SignalP [43]. Both MUSCLE [44] and Probcons [45] were employed for multiple protein sequence alignments, with manual editing to improve alignments of the repeating LRRs and coiled-coils. These alignments were used to build neighbour joining phylogenetic trees with ClustalW [46] to predict the likely evolutionary relations among the genes supported by gene orthology assignments from the OrthoDB resource [47]. Protein sequence logos [48] were built from the LRR alignments to examine amino acid conservation patterns. The relative genomic organisations of the identified *LRIM*-like genes in each of the three mosquito species were examined using the VectorBase [49] genome browser facilities. Perl regular expressions were developed to identify and measure the lengths of individual LRRs of the LRIM-like proteins and to scan the predicted protein-coding genes of each mosquito species as well as those of *A. mellifera*, *Bombyx mori *(silk moth), *D. melanogaster*, and *Tribolium castaneum *(flour beetle). The VectorBase expression data BioMart facility (database release 1.1.2) was employed to identify *An. gambiae *LRIMs that exhibited transcriptional responses to malaria parasite infections or blood feeding.

### PCR Analysis of PANGGL Repeat Region of *AgAPL1C*

Genomic DNA was prepared from single male mosquitoes from three laboratory strains; G3, L3-5 and Yaounde. Mosquitoes were placed in a tube containing 100 μL of a 50% w/v suspension of Chelex 100 beads (Sigma) in water. Mosquitoes and beads were homogenised using a disposable pestle and samples were incubated at 100°C for 10 minutes. Homogenates were spun at 20,000 xg for 1 minute. 1 μL of an 800 unit/mL stock of Proteinase K (Sigma) was added to 10 μL of the cleared supernatant and the samples were incubated at 37°C for 30 minutes, and then at 100°C for 5 minutes. 10 uL PCR reactions were run using 0.5 μL of the genomic DNA, GoTaq master mix (Promega) and the following primers flanking the *AgAPL1C *PANGGL repeat region: gcggatccaccATGTGCTGGTTACACGCCGTATC and ACCTATATGGGTTGGAGTTC. Products were analysed on a 2% agarose gel.

## Abbreviations

APL: *Anopheles-Plasmodium*-responsive leucine-rich repeat protein; BRACA: breast cancer susceptibility protein; cDNA: complementary deoxyribonucleic acid; GNEF: guanine nucleotide exchange factor; LRIM: leucine-rich repeat immune protein; LRL: leucine-rich leader; LRR: leucine-rich repeat; MD: myeloid differentiation protein; NBD: nucleotide-binding domain; NLR: nucleotide-binding leucine-rich repeat protein; PAMP: pathogen associated molecular pattern; SNARE: soluble N-ethylmaleimide-sensitive factor attachment protein receptor; TE: transposable element; TEP: thioester-containing protein; TIR: toll-interleukin receptor; TLR: toll-like receptor; TM: transmembrane; VLR: variable lymphocyte receptor.

## Competing interests

The authors declare that they have no competing interests.

## Authors' contributions

RMW conceived and designed the study, carried out the computational comparative genomic analyses, and wrote the manuscript. MP performed the PANGGL repeat polymorphism experiments, participated in the design of the study and helped to write the manuscript. GKC coordinated the study, participated in the design, and helped to write the manuscript. All authors read and approved the final manuscript.

## Supplementary Material

Additional file 1 **Supplementary tables of *LRIM *gene features and expression data**. A table listing the sequence features of each of the identified *LRIM *genes in the mosquitoes *Anopheles gambiae*, *Aedes aegypti *and *Culex quinquefasciatus*, and a table listing *Anopheles gambiae LRIM *genes with experimental evidence of transcriptional responses to malaria parasite infections or blood feeding. Click here for file
