# Supplementary material for: Sequence-structure-function relations of the mosquito leucine-rich repeat immune proteins
Source: BMC Genomics. 2010 Sep 30;11:531. doi: 10.1186/1471-2164-11-531 (PMC3020904; doi:10.1186/1471-2164-11-531)
Supplement: Additional file 1 — Supplementary tables of LRIM gene features and expression data. A table listing the sequence features of each of the identified LRIM genes in the mosquitoes Anopheles gambiae, Aedes aegypti and Culex quinquefasciatus, and a table listing Anopheles gambiae LRIM genes with experimental evidence of transcriptional responses to malaria parasite infections or blood feeding. [file 1471-2164-11-531-S1.PDF]

Additional file 1

Supplementary Tables 1 and 2.

Sequence-structure-function relations of the mosquito leucine-rich repeat immune proteins

Robert M Waterhouse, Michael Povelones, and George K Christophides

Supplementary Table 1.

LRIM family members in *Anopheles gambiae* (AGAP), *Aedes aegypti* (AAEL) and *Culex quinquefasciatus* (CPIJ). LRIM types include the Long (L), Short (S), Transmembrane (TM) and Coil-less (CL) subfamilies. Alternative names (Alt. Name) with corresponding references are given for genes described previously or for split official gene models.

SP signal peptide.
C-C N-terminal cysteine motif.
LRL leucine-rich leader sequence.
LRRs number of identified leucine-rich repeats.
C-CC C-terminal double-cysteine motif.
C* C-terminal single cysteine.
C-coils number of coiled-coil regions [Marcoil, (Delorenzi and Speed, 2002)].
√ sequence feature is present.
X sequence feature is absent.
¤ less than 90% coiled-coil probability [Marcoil, (Delorenzi and Speed, 2002)].
° LRIM28, and possibly LRIM22, may be considered as Long LRIMs due to their putative coiled-coil regions and 10 LRRs, however sequence clustering suggests they are more closely-related to the Coil-less LRIMs.
References: (1) (Osta, et al., 2004), (2) (Riehle, et al., 2008), (3) (Riehle, et al., 2006), (4) (Habtewold, et al., 2008), (5) (Dong, et al., 2006), (6) (Aguilar, et al., 2005).

Type	Gene-ID or Location	LRIM	Alt. Name	SP	C-C	LRL	LRRs	C-CC	C*	C-coils
L	AGAP006348	*LRIM1(1)*		√	*X*	*X*	10	√	√	2
L	AAEL012086	*LRIM1*		√	√	*X*	10	*X*	√	2
L	CPIJ004924	*LRIM1*		√	√	*X*	10	√	√	2
L	AGAP007036	*APL1A(2)*	*APL1(3)* *LRIM2(4)*	√	√	√	12	*X*	√	2
L	AGAP007035	*APL1B(2)*	*APL1(3)* *LRIM2(4)*	√	*X*	√	12	√	√	2
L	AGAP007033	*APL1C(2)*	*APL1(3)* *LRIM2(4)* *LRRD19(5)*	√	√	√	12	√	√	2
L	AAEL009520	*LRIM2*		√	√	√	13	√	√	2
L	CPIJ002970	*LRIM2A*		√	√	√	13	√	√	2(1¤)
L	CPIJ002973	*LRIM2B*		√	√	√	13	√	√	2
L	CPIJ002975	*LRIM2C*		*X*	√	√	13	√	√	2
L	AGAP007037	*LRIM3*		√	√	√	13	√	*X*	1
L	AAEL010132	*LRIM3*		√	√	√	13	√	*X*	1
L	CPIJ012895	*LRIM3*		√	√	√	13	√	*X*	1
L	AGAP007039	*LRIM4*	*LRRD5(5)*	√	√	√	11	√	*X*	2
L	AAEL010128	*LRIM4*		√	√	√	11	√	*X*	1
L	CPIJ012900	*LRIM4*		√	√	√	11	√	*X*	1
S	Ag2R: 17513769-4965	*LRIM5*		√	√	√	6	√	*X*	1
S	AAEL012767	*LRIM5*		√	√	√	6	√	*X*	1
S	CPIJ017195	*LRIM5*		√	√	√	6	√	*X*	2
S	AGAP006327	*LRIM6*		√	√	√	6	√	*X*	1
S	AAEL012538	*LRIM6*		√	√	√	6	√	*X*	1
S	CPIJ008240	*LRIM6*		√	√	√	6	√	*X*	1
S	AGAP007457	*LRIM7*	*LRRD17(5)*	√	√	√	7	√	*X*	1
S	AAEL001417	*LRIM7*		√	√	√	7	√	*X*	1
S	CPIJ017602	*LRIM7*		√	√	√	7	√	*X*	1
S	AGAP007454	*LRIM8A*		√	√	√	7	√	*X*	2
S	AGAP007456	*LRIM8B*	*LRRD4(5)* *LRRd-1(6)*	√	√	√	7	√	*X*	2
S	AAEL001420	*LRIM8*		√	√	√	7	√	*X*	1
S	CPIJ017601	*LRIM8*		√	√	√	7	√	*X*	3
S	AGAP007453	*LRIM9*		√	√	√	7	√	*X*	1
S	AAEL001414	*LRIM9*		√	√	√	7	√	*X*	1
S	CPIJ017599	*LRIM9*		√	√	√	7	√	*X*	1
S	AGAP007455	*LRIM10*	*LRRD9(5)*	√	√	√	7	√	*X*	2(1¤)
S	AAEL001401	*LRIM10A*		√	√	√	7	√	*X*	2
S	AAEL001402	*LRIM10B*		√	√	√	7	√	*X*	2
S	CPIJ017600	*LRIM10*		√	√	√	7	√	*X*	2
S	AGAP007034	*LRIM11*		√	√	√	7	√	*X*	1
S	AAEL010129	*LRIM11*		√	√	√	7	√	*X*	1
S	CPIJ012896	*LRIM11*		√	√	√	7	√	*X*	1
S	AGAP005496	*LRIM12*		√	√	√	7	√	*X*	2
S	AAEL010656	*LRIM12*		√	√	√	7	√	*X*	2
S	CPIJ005661	*LRIM12*		√	√	√	7	√	*X*	2
S	AAEL012255	*LRIM13*		√	√	√	7	√	*X*	1
S	CPIJ016345	*LRIM13*		√	√	√	7	√	*X*	1
S	CPIJ004912	*LRIM14*		√	√	√	7	√	*X*	1¤
TM	AGAP007045	*LRIM15*		√	√	√	13	C-YC	*X*	1
TM	AAEL007103	*LRIM15*		√	√	√	13	C-YC	*X*	1¤
TM	CPIJ013628	*LRIM15*		√	√	√	13	C-YC	*X*	1¤

Type	Gene-ID or Location	LRIM	Alt. Name	SP	C-C	LRL	LRRs	C-CC	C*	C-coils
TM	Ag3R: 317336-9560	*LRIM16A*	*AGAP007758*	√	√	√	14	C-YC	*X*	1¤
TM	Ag3R: 322657-4842	*LRIM16B*	*AGAP007758*	√	√	√	14	C-YC	*X*	1¤
TM	AAEL008658	*LRIM16*		√	√	√	14	-YC	*X*	1
TM	CPIJ006267	*LRIM16*		√	√	√	14	-YC	*X*	1
CL	AGAP005693	*LRIM17*	*APL2(3)* *LRRD7(5)*	√	√	√	13	√	*X*	0
CL	AAEL010125	*LRIM17*		√	√	√	13	√	*X*	0
CL	CPIJ012894	*LRIM17*		√	√	√	13	√	*X*	0
CL	AGAP010675	*LRIM18*		√	√	√	7	√	*X*	0
CL	AAEL012911	*LRIM18*		√	√	√	7	√	*X*	0
CL	CPIJ005020	*LRIM18*		√	√	√	7	√	*X*	0
CL	AGAP011117	*LRIM19*		√	√	√	7	√	*X*	0
CL	AAEL000762	*LRIM19*		√	√	√	7	√	*X*	0
CL	CPIJ010209	*LRIM19*		√	√	√	7	√	*X*	0
CL	AGAP002542	*LRIM20*		√	*X*	√	6	√	*X*	0
CL	AAEL006920	*LRIM20*		√	*X*	√	6	√	*X*	0
CL	CPIJ009595	*LRIM20*		√	*X*	√	6	√	*X*	0
CL	AAEL009894	*LRIM21*		√	√	√	7	√	*X*	0
CL	CPIJ007509	*LRIM21*		√	√	√	7	√	*X*	0
CL°	AAEL007224	*LRIM22*		√	√	√	10	√	*X*	1¤
CL°	CPIJ015874	*LRIM22*		√	√	√	10	√	*X*	1¤
CL	AAEL004466	*LRIM23*		√	√	√	6	√	*X*	0
CL	CPIJ014357	*LRIM23*		√	√	√	6	√	*X*	0
CL	AAEL012763	*LRIM24*		√	√	√	6	√	*X*	0
CL	CPIJ017196	*LRIM24*		√	√	√	6	√	*X*	0
CL	AAEL009792	*LRIM25*		√	√	√	7	√	*X*	0
CL	CPIJ000810	*LRIM25*		√	√	√	7	√	*X*	0
CL	AGAP005744	*LRIM26*		√	√	√	7	√	*X*	0
CL	Ag2R: 17512465-3625	*LRIM27*		√	√	√	6	√	*X*	0
CL°	AAEL007231	*LRIM28*		√	√	√	10	√	*X*	1
CL°	CPIJ009406	*LRIM28*		√	√	√	10	√	*X*	1
CL	AAEL015627	*LRIM29*		√	√	√	6	√	*X*	0
CL	AAEL012771	*LRIM30*		√	√	√	6	√	*X*	0
CL	AAEL006377	*LRIM31*		√	√	√	7	√	*X*	0
CL	CPIJ011510	*LRIM32*		√	√	√	7	√	*X*	0
CL	CPIJ017197	*LRIM33*		√	√	√	6	√	*X*	0

Supplementary Table 2.

Anopheles gambiae LRIM family members with experimental evidence of transcriptional responses to malaria parasite infections or blood feeding. Data were retrieved from the VectorBase (Lawson, et al., 2009) expression data BioMart facility (database release 1.1.2), see www.vectorbase.org for complete datasets. The experimental details for the parasite infections are described in (Vlachou, et al., 2005) and for blood feeding in (Marinotti, et al., 2005).

LRIM	Gene ID	Experiment Name	Statistical Test	P-Value
APL1A	AGAP007036	Blood meal time series	ANOVA	6.41E-07
	Significant differential expression, with highest expression in GrowthCondition = Blood-fed 3h and lowest expression in GrowthCondition = Blood-fed 72h			
APL1A	AGAP007036	Blood-fed adult female tissues	ANOVA	4.18E-06
	Significant differential expression, with highest expression in OrganismPart = fat body [TGMA:0001856] and lowest expression in OrganismPart = midgut [TGMA:0001036]			
APL1C	AGAP007033	Blood meal time series	ANOVA	6.41E-07
	Significant differential expression, with highest expression in GrowthCondition = Blood-fed 3h and lowest expression in GrowthCondition = Blood-fed 72h			
APL1C	AGAP007033	Blood-fed adult female tissues	ANOVA	4.18E-06
	Significant differential expression, with highest expression in OrganismPart = fat body [TGMA:0001856] and lowest expression in OrganismPart = midgut [TGMA:0001036]			
LRIM1	AGAP006348	Blood meal time series	ANOVA	5.88E-05
	Significant differential expression, with highest expression in GrowthCondition = Blood-fed 3h and lowest expression in GrowthCondition = Blood-fed 72h			
LRIM1	AGAP006348	Blood-fed adult female tissues	ANOVA	1.36E-07
	Significant differential expression, with highest expression in OrganismPart = fat body [TGMA:0001856] and lowest expression in OrganismPart = ovaries			
LRIM1	AGAP006348	*Plasmodium berghei* midgut invasion stage comparisons	t-test	8.51E-04
	Significant 2.5-fold up-regulation with respect to DiseaseState = wild-type parasite infection; DiseaseStaging = during midgut invasion v before midgut invasion			
LRIM1	AGAP006348	*Plasmodium berghei* midgut invasion time-series	t-test	2.70E-04
	Significant 2.2-fold up-regulation with respect to DiseaseState = wild-type parasite infection v invasion-deficient parasite infection; DiseaseStaging = during midgut invasion			
LRIM3	AGAP007037	Blood meal time series	ANOVA	6.01E-08
	Significant differential expression, with highest expression in GrowthCondition = Blood-fed 96h and lowest expression in GrowthCondition = Blood-fed 72h			
LRIM3	AGAP007037	Blood-fed adult female tissues	ANOVA	6.53E-03
	Significant differential expression, with highest expression in OrganismPart = fat body [TGMA:0001856] and lowest expression in OrganismPart = ovaries			
LRIM4	AGAP007039	Blood meal time series	ANOVA	8.25E-07
	Significant differential expression, with highest expression in GrowthCondition = Blood-fed 96h and lowest expression in GrowthCondition = Blood-fed 24h			
LRIM4	AGAP007039	Blood-fed adult female tissues	ANOVA	2.93E-08
	Significant differential expression, with highest expression in OrganismPart = fat body [TGMA:0001856] and lowest expression in OrganismPart = ovaries			
LRIM4	AGAP007039	*Plasmodium berghei* midgut invasion stage comparisons	t-test	1.09E-04
	Significant 1.4-fold up-regulation with respect to DiseaseState = invasion-deficient parasite infection; DiseaseStaging = during midgut invasion v before midgut invasion			
LRIM4	AGAP007039	*Plasmodium berghei* midgut invasion time-series	t-test	2.64E-03
	Significant 1.3-fold down-regulation with respect to DiseaseState = wild-type parasite infection v invasion-deficient parasite infection; DiseaseStaging = during midgut invasion			
LRIM6	AGAP006327	Blood meal time series	Neighbour t-test	2.00E-05
	Significant upward change in expression, between GrowthCondition = Blood-fed 72h and GrowthCondition = Blood-fed 96h			
LRIM6	AGAP006327	Blood-fed adult female tissues	ANOVA	5.50E-07
	Significant differential expression, with highest expression in OrganismPart = midgut [TGMA:0001036] and lowest expression in OrganismPart = ovaries			
LRIM6	AGAP006327	*Plasmodium berghei* midgut invasion stage comparisons	t-test	4.54E-02
	Significant 2.1-fold down-regulation with respect to DiseaseState = invasion-deficient parasite infection; DiseaseStaging = after midgut invasion v during midgut invasion			

LRIM	Gene ID	Experiment Name	Statistical Test	P-Value
LRIM6	AGAP006327	*Plasmodium berghei* midgut invasion time-series	t-test	1.30E-02
	Significant 1.5-fold down-regulation with respect to DiseaseState = wild-type parasite infection v invasion-deficient parasite infection; DiseaseStaging = before midgut invasion			
LRIM7	AGAP007457	Blood meal time series	ANOVA	2.50E-06
	Significant differential expression, with highest expression in GrowthCondition = Blood-fed 96h and lowest expression in GrowthCondition = Blood-fed 15d			
LRIM7	AGAP007457	Blood-fed adult female tissues	ANOVA	1.04E-04
	Significant differential expression, with highest expression in OrganismPart = fat body [TGMA:0001856] and lowest expression in OrganismPart = ovaries			
LRIM8A	AGAP007454	*Plasmodium berghei* midgut invasion time-series	t-test	3.25E-04
	Significant 1.8-fold up-regulation with respect to DiseaseState = wild-type parasite infection v invasion-deficient parasite infection; DiseaseStaging = during midgut invasion			
LRIM8B	AGAP007456	Blood meal time series	ANOVA	4.38E-11
	Significant differential expression, with highest expression in GrowthCondition = Blood-fed 24h and lowest expression in GrowthCondition = Blood-fed 3h			
LRIM8B	AGAP007456	Blood-fed adult female tissues	ANOVA	7.42E-09
	Significant differential expression, with highest expression in OrganismPart = fat body [TGMA:0001856] and lowest expression in OrganismPart = ovaries			
LRIM8B	AGAP007456	*Plasmodium berghei* midgut invasion stage comparisons	t-test	1.85E-02
	Significant 1.3-fold down-regulation with respect to DiseaseState = invasion-deficient parasite infection; DiseaseStaging = after midgut invasion v during midgut invasion			
LRIM9	AGAP007453	Blood meal time series	ANOVA	6.29E-13
	Significant differential expression, with highest expression in GrowthCondition = Blood-fed 24h and lowest expression in GrowthCondition = Blood-fed 3h			
LRIM9	AGAP007453	Blood-fed adult female tissues	ANOVA	2.99E-09
	Significant differential expression, with highest expression in OrganismPart = fat body [TGMA:0001856] and lowest expression in OrganismPart = ovaries			
LRIM10	AGAP007455	Blood meal time series	ANOVA	1.97E-14
	Significant differential expression, with highest expression in GrowthCondition = Blood-fed 24h and lowest expression in GrowthCondition = Blood-fed 3h			
LRIM10	AGAP007455	Blood-fed adult female tissues	ANOVA	2.23E-08
	Significant differential expression, with highest expression in OrganismPart = fat body [TGMA:0001856] and lowest expression in OrganismPart = ovaries			
LRIM10	AGAP007455	*Plasmodium berghei* midgut invasion stage comparisons	t-test	7.23E-03
	Significant 2.2-fold up-regulation with respect to DiseaseState = wild-type parasite infection; DiseaseStaging = during midgut invasion v before midgut invasion			
LRIM10	AGAP007455	*Plasmodium berghei* midgut invasion time-series	t-test	1.65E-04
	Significant 2.7-fold up-regulation with respect to DiseaseState = wild-type parasite infection v invasion-deficient parasite infection; DiseaseStaging = during midgut invasion			
LRIM12	AGAP005496	Blood meal time series	ANOVA	5.56E-05
	Significant differential expression, with highest expression in GrowthCondition = Blood-fed 15d and lowest expression in GrowthCondition = Blood-fed 24h			
LRIM15	AGAP007045	Blood meal time series	Neighbour t-test	3.63E-03
	Significant upward change in expression, between GrowthCondition = Blood-fed 24h and GrowthCondition = Blood-fed 48h			
LRIM15	AGAP007045	Blood-fed adult female tissues	ANOVA	6.94E-04
	Significant differential expression, with highest expression in OrganismPart = fat body [TGMA:0001856] and lowest expression in OrganismPart = ovaries			
LRIM16A/B	AGAP007758	Blood meal time series	Neighbour t-test	1.84E-04
	Significant upward change in expression, between GrowthCondition = Blood-fed 3h and GrowthCondition = Blood-fed 24h			
LRIM16A/B	AGAP007758	Blood-fed adult female tissues	ANOVA	1.39E-06
	Significant differential expression, with highest expression in OrganismPart = fat body [TGMA:0001856] and lowest expression in OrganismPart = ovaries			
LRIM17	AGAP005693	Blood meal time series	Neighbour t-test	1.71E-03
	Significant upward change in expression, between GrowthCondition = Blood-fed 24h and GrowthCondition = Blood-fed 48h			
LRIM17	AGAP005693	Blood-fed adult female tissues	ANOVA	8.21E-09
	Significant differential expression, with highest expression in OrganismPart = fat body [TGMA:0001856] and lowest expression in OrganismPart = ovaries			
LRIM19	AGAP011117	Blood meal time series	Neighbour t-test	5.27E-03
	Significant upward change in expression, between GrowthCondition = Blood-fed 72h and GrowthCondition = Blood-fed 96h			
LRIM19	AGAP011117	Blood-fed adult female tissues	ANOVA	3.59E-02
	Significant differential expression, with highest expression in OrganismPart = midgut [TGMA:0001036] and lowest expression in OrganismPart = ovaries			

LRIM	Gene ID	Experiment Name	Statistical Test	P-Value
LRIM20	AGAP002542	Blood meal time series	Neighbour t-test	8.84E-03
	Significant upward change in expression, between GrowthCondition = Blood-fed 24h and GrowthCondition = Blood-fed 48h			
LRIM20	AGAP002542	Blood-fed adult female tissues	ANOVA	1.23E-03
	Significant differential expression, with highest expression in OrganismPart = midgut [TGMA:0001036] and lowest expression in OrganismPart = ovaries			
LRIM26	AGAP005744	Blood meal time series	ANOVA	3.22E-08
	Significant differential expression, with highest expression in GrowthCondition = Blood-fed 24h and lowest expression in GrowthCondition = Non-blood-fed			
LRIM26	AGAP005744	*Plasmodium berghei* midgut invasion time-series	t-test	6.80E-05
	Significant 1.9-fold up-regulation with respect to DiseaseState = wild-type parasite infection v invasion-deficient parasite infection; DiseaseStaging = during midgut invasion			

References

Aguilar, R, AE Jedlicka, M Mintz, V Mahairaki, AL Scott, and G Dimopoulos. 'Global Gene Expression Analysis of Anopheles Gambiae Responses to Microbial Challenge.', *Insect Biochem Mol Biol* **Vol. 35, No. 7**, 709-19, 2005.

Delorenzi, M, and T Speed. 'An Hmm Model for Coiled-Coil Domains and a Comparison with Pssm-Based Predictions.', *Bioinformatics* **Vol. 18, No. 4**, 617-25, 2002.

Dong, Y, R Aguilar, Z Xi, E Warr, E Mongin, and G Dimopoulos. 'Anopheles Gambiae Immune Responses to Human and Rodent Plasmodium Parasite Species.', *PLoS Pathog* **Vol. 2, No. 6**, e52, 2006.

Habtewold, T, M Povelones, AM Blagborough, and GK Christophides. 'Transmission Blocking Immunity in the Malaria Non-Vector Mosquito Anopheles Quadriannulatus Species A.', *PLoS Pathog* **Vol. 4, No. 5**, e1000070, 2008.

Lawson, D, P Arensburger, P Atkinson, NJ Besansky, RV Bruggner, R Butler, KS Campbell, GK Christophides, S Christley, E Dialynas, M Hammond, CA Hill, N Konopinski, NF Lobo, RM MacCallum, G Madey, K Megy, J Meyer, S Redmond, DW Severson, EO Stinson, P Topalis, E Birney, WM Gelbart, FC Kafatos, C Louis, and FH Collins. 'Vectorbase: A Data Resource for Invertebrate Vector Genomics.', *Nucleic Acids Res* **Vol. 37, No. Database issue**, D583-7, 2009.

Marinotti, O, QK Nguyen, E Calvo, AA James, and JM Ribeiro. 'Microarray Analysis of Genes Showing Variable Expression Following a Blood Meal in Anopheles Gambiae.', *Insect Mol Biol* **Vol. 14, No. 4**, 365-73, 2005.

Osta, MA, GK Christophides, and FC Kafatos. 'Effects of Mosquito Genes on Plasmodium Development.', *Science* **Vol. 303, No. 5666**, 2030-2, 2004.

Riehle, MM, K Markianos, O Niaré, J Xu, J Li, AM Touré, B Podiougou, F Oduol, S Diawara, M Diallo, B Coulibaly, A Ouatara, L Kruglyak, SF Traoré, and KD Vernick. 'Natural Malaria Infection in Anopheles Gambiae Is Regulated by a Single Genomic Control Region.', *Science* **Vol. 312, No. 5773**, 577-9, 2006.

Riehle, MM, J Xu, BP Lazzaro, SM Rottschaefer, B Coulibaly, M Sacko, O Niare, I Morlais, SF Traore, and KD Vernick. 'Anopheles Gambiae Apl1 Is a Family of Variable Lrr Proteins Required for Rel1-Mediated Protection from the Malaria Parasite, Plasmodium Berghei.', *PLoS One* **Vol. 3, No. 11**, e3672, 2008.

Vlachou, D, T Schlegelmilch, GK Christophides, and FC Kafatos. 'Functional Genomic Analysis of Midgut Epithelial Responses in Anopheles During Plasmodium Invasion.', *Curr Biol* **Vol. 15, No. 13**, 1185-95, 2005.
